# Challenging the current dogma of chronic Cd nephrotoxicity: myths and facts

**DOI:** 10.1007/s00204-025-04264-0

**Published:** 2026-01-14

**Authors:** Frank Thévenod, Wing-Kee Lee

**Affiliations:** 1 Institute for Physiology and Pathophysiology ZBAF, Witten/Herdecke University, 58453 Witten, Germany; 2 Physiology and Pathophysiology of Cells and Membranes, Medical School OWL, Bielefeld University, 33615 Bielefeld, Germany

Renal damage was first linked to occupational cadmium (Cd) exposure in humans by L. Friberg in 1948. Innumerable studies since have evidenced Cd bound to metallothionein (MT), its accumulation in the renal proximal tubule (PT), and subsequent cellular injury. Many toxicologists assume the current model of chronic Cd nephrotoxicity (chrCd-NTX), in which filtered Cd-MT plays an eminent role in Cd transport from the liver to the kidneys, is valid and exhaustive, and further research on renal Cd uptake and toxicity is considered superfluous (Nordberg and Nordberg 2025). The Nordberg model was established more than 50 years ago (reviewed in (Nordberg and Nordberg 2025)) based on studies of acute nephrotoxicity. Remarkably, this model has prevailed for chrCd-NTX despite solid evidence of other Cd-interacting blood constituents and Cd handling by specialized cells of the liver and kidneys.

In our opinion, based on evidence-based reasoning, circulating Cd-MT is, in fact, very unlikely to substantially contribute to Cd-induced PT damage. Hence, we request the Cd community to consider, in earnest, the *status quo* of the mechanisms for hepato-renal Cd transfer, importance of Cd speciation and necessity for a more nuanced understanding of circulating Cd-complexes in the blood, which dictate their renal damaging capacity.

## Cd in the environment

From the Earth’s crust, Cd is released naturally by volcanic emissions, forest fires and weathering of rocks, and exists preferentially in one oxidation state (+2). Anthropogenic sources elevate Cd levels. Over time, non-biodegradable Cd in the environment increments steadily and globally. Cd is ranked seventh on the Hazardous Substances Priority List and implementation of strict safety regulations prevent recurrence of health hazards in exposed populations.

### Why should we still care about Cd?

Not because of acute Cd intoxication nor in an occupational context, but because Cd toxicity becomes increasingly relevant for global human, animal and plant health as environmental Cd levels rise continuously. The real challenge we face in the twenty-first century is chronic low Cd exposure (CLCE), primarily from contaminated dietary sources and tobacco usage. In fact, CLCE is a significant health hazard for ~ 10% of the world’s population that increases morbidity and mortality (Moulis and Thévenod 2010). Worryingly, without a known threshold, there currently appears to be no safe limit for CLCE.

## Cd in the human body

Following Cd ingestion or inhalation, various Cd metal species are formed in the blood and reach the liver, which removes potentially toxic compounds. The Cd ion (Cd^2+^) forms complexes with metalloproteins (e.g. MT), zinc-finger, iron-binding, low- and high molecular weight plasma proteins as well as with physiological small organic molecules, such as glutathione.

### Do we know which Cd-protein complexes prevail in blood?

In short, no. The formation of Cd-protein complexes is inferred from affinity to Cd and in vivo protein abundance (see Suppl. Table 1 for details). When Cd (0.1–1 µM) is added ex vivo to serum proteins, unsurprisingly, it is bound to abundant Alb but also to alpha-2-macroglobulin and immunoglobulins (Ig) (Scott and Bradwell 1983). In Alb/IgG-depleted blood samples, apolipoprotein A-I (ApoA1) was identified as a major Cd-binding protein in non-occupational human donors ([Cd] = 0.08 ± 0.07 ng/ml) (Li et al. 2020).

## The Nordberg model

The prevailing model for chrCd-NTX (Nordberg and Nordberg 2025) was developed in the 1960–90s from animal studies using acute single intravenous injections of high CdCl_2_ or Cd-MT, motivated by high occupational or environmental Cd and are reasonable models of acute/subacute Cd nephrotoxicity. Since injected Cd-MT in mice is quickly cleared from the plasma by the kidneys (Nordberg and Nordberg 2025), MT in the circulation was thought to be essential for Cd delivery to the kidney.

**Fig. 1:**
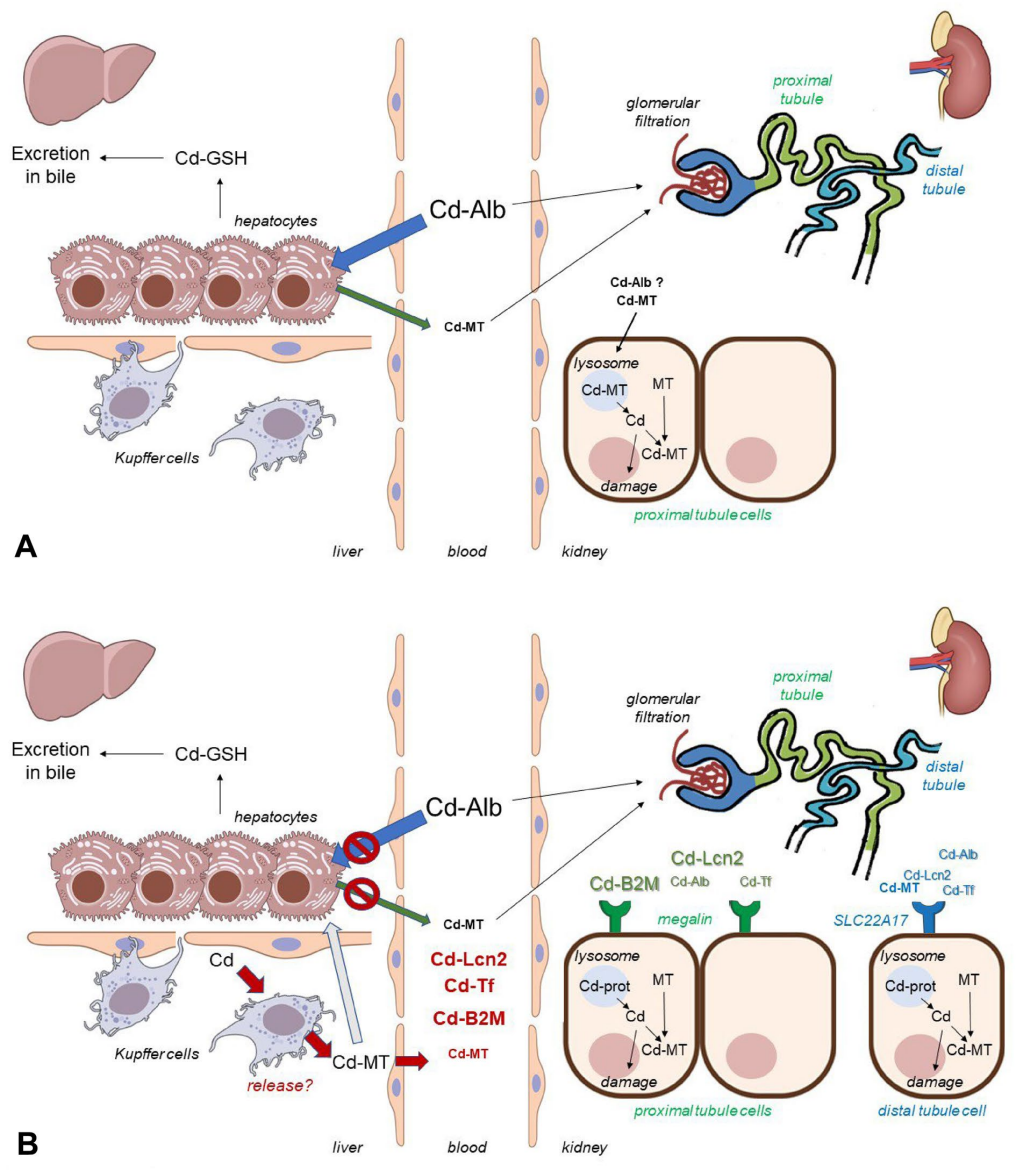
**A** Nordberg model of Cd fate in the body demonstrating the role of binding forms in blood, and MT synthesis and toxicity in the kidney proximal tubule (derived from (Nordberg and Nordberg 2025)). **B** Proposed revised model of Cd fate in the body demonstrating the role of transport by binding-proteins in blood, uptake by receptor-mediated endocytosis and toxicity in kidney proximal and distal tubule cells. Illustrations partly from NIAID NIH BioArt Source (bioart.niaid.nih.gov/bioart). For further details, see text

The Nordberg model assumes: Cd bound to plasma Alb is taken up by liver hepatocytes, wherein cytosolic Cd combines with GSH and is excreted into bile, or binds to intracellular MT, creating a storage form of Cd. Some Cd-MT “leaks” into the plasma, thus entering the systemic circulation, presumably consequent of necrotic hepatocytes. Following ultrafiltration in the kidney, Cd-MT is endocytosed by PT cells, entering lysosomes, wherein MT is degraded to amino acids, and Cd is released into the cytosol to cause cellular injury, or is chelated by renal MT. At concentrations exceeding 200 µg/g Cd tissue, kidney damage and proteinuria ensue (see Fig. 1A).

## Flaws of the Nordberg model

The plausibility of the Nordberg model for chrCd-NTX suffers from several conceptual and methodological shortcomings.

- **Here we lay the reasoning for our opinion**
  1. Animal models of kidney injury employing an acute bolus injection of CdCl_2_ or Cd-MT into the circulation are too harsh and do not recapitulate the subtle changes observed in humans, which occur at Cd concentrations as low as 50 µg/g tissue (Thijssen et al. 2007). Thus, chrCd-NTX studies in whole animals need to be redefined.
  2. No in vivo experimental data describe uptake and entry pathways for Cd-complexes in liver cells. In primary hepatocytes, Cd-MT rather than Cd-Alb is taken up (Pham et al. 2004), which does not support the Nordberg model (Nordberg and Nordberg 2025; Figure 1A). Moreover, Kupffer cells and liver sinusoidal endothelial cells harbor elevated endogenous MT compared to hepatocytes (Caperna and Failla 1984) and can accumulate more Cd (McKim et al. 1992), indicating they are “Cd defense” cells.
  3. Cd-MT leakage as a complex from damaged hepatocytes into plasma in animals (Nordberg and Nordberg 2025; Figure 1A) or into extracellular medium from cultured cells has never been demonstrated. Only liver or kidney Cd or MT (Chan et al. 1993) have been measured; this is correlative and there is no substantiate evidence that Cd-MT is liberated as an intact complex, and questions Cd-MT as the major transport form to the kidneys in plasma.
  4. Despite its high affinity to Cd, plasma MT abundance (~ 0.5 to 5 nM) is very low compared to other filtered plasma proteins, such as Alb (~ 0.7 mM), transferrin (Tf) (~ 35 µM), lipocalin-2/NGAL/24p3 (LCN2) (7 µM), alpha-1-microglobulin (~ 1 µM) or beta-2-microglobulin (β2M); ~ 0.1 µM), suggesting they are more likely candidates of Cd metalation.
  5. MT binding to the principal protein endocytic receptor megalin in the PT occurs at a single site with a *Kd* of ~ 10^−4^ M (see Suppl. Table 2A for details). Given megalin’s affinity to MT/Cd-MT is ~ 10^5^-times lower than MT in the glomerular filtrate, it is highly unlikely to bind and endocytose filtered MT/Cd-MT into the PT in vivo. Hence, filtered Cd-MT as the nephrotoxic Cd species is doubtful.
  6. Finally, the most convincing argument is derived from studies using MT-knockout mice (Liu et al. 1998). Although renal Cd burden and renal MT were far higher in wildtype mice than MT-knockout mice, the maximally tolerated dose of Cd in MT-knockout mice was approximately one-eighth that of controls, indicating MT is protective against—rather than mediating—chrCd-NTX (Liu et al. 1998).

## Handling of circulating Cd-protein complexes by the kidneys: facts and controversies

To reach renal tubular epithelial cells, Cd-protein complexes must first undergo ultrafiltration. Permeation of the glomerular filtration barrier decreases with increasing molecular mass (MM) (cutoff ~ 80 kDa), molecular size (< 42Å), and negative charge. Proteins (and their Cd-protein complexes), such as MT (MM ~ 7 kDa), β2M (MM ~ 12 kDa), ApoA1 (MM ~ 28 kDa), and LCN2 (MM ~ 25 kDa) are either freely or significantly filtered by the glomerulus, whereas a small but significant proportion of Alb (MM 65 kDa) crosses the glomerular barrier, reaching ~ 50 nM in the glomerular filtrate (reviewed in Thévenod et al. 2023; Thévenod and Wolff 2016)). In contrast, alpha-2-macroglobulin (MM ~ 720 kDa) is size excluded. Thus, not all Cd-binding serum proteins reach tubular cells, deeming them irrelevant for chrCd-NTX.

### Why is the renal PT targeted by Cd?

The PT is responsible for bulk reabsorption of primary urine and possesses numerous apical uptake mechanisms, which also largely contribute to the reabsorption of Cd (reviewed in (Thévenod and Wolff 2016)). Apart from fluid phase endocytosis, the abundance of filtered proteins in the tubular lumen dictates protein/Cd-protein reabsorption. Supposing the current hypothesis of chrCd-NTX is valid, by which mechanism(s) can Cd-MT enter PT cells? The most obvious candidate would be the megalin/cubilin receptor complex, which recovers filtered plasma proteins. Even though MT is freely filtered, MT concentrations in the glomerular filtrate are insufficient to bind and initiate internalization by megalin/cubilin in vivo; even in Cd-elevated states, such as smokers, where plasma MT reaches ~ 5 nM.

Of course, megalin does recognize MT bound to Cd; this we do not doubt. Studies in cell culture and mouse models, essentially as proof of principle, showed uptake of micromolar Cd-MT by megalin/cubulin (Onodera et al. 2012), which is trafficked to acidic lysosomes wherein MT is degraded and ionic Cd exits via divalent metal transporter-1 (DMT1/SLC11A2) into the cytosol (Abouhamed et al. 2007). These mechanisms of metal trafficking are physiologically relevant for essential metals and their protein complexes, but not for Cd-MT, which circulates at concentrations at least 1000-fold lower in plasma and glomerular filtrate than the experimental micromolar Cd-MT concentrations used (see Suppl. Tables 1 and 2 for details).

### If Cd-MT is not internalized by the PT, why is Cd-MT found in the kidney?

A fundamental misconception of the Nordberg model for chrCd-NTX lies in its understanding of “transport” versus “storage” roles of (Cd-)MT in the kidney (Nordberg and Nordberg 2025). Low Cd stress stimulates PT cells to upregulate MT, which chelates “free” Cd for long-term storage in the cytosol. When wildtype and MT-knockout mice were administered with ^109^CdCl_2_ (15 µmol/kg, i.p.) (Liu et al. 1996), renal Cd increased to a similar extent between 2 h and 2 days, and remained elevated for up to 15 days in wildtype mice, but decreased in MT-null mice. Similar observations were made in another study where kidneys of MT-null mice accumulated 14-times less Cd than wildtype kidneys after 3–6 weeks subcutaneous CdCl_2_ injections (Liu et al. 1998). Conclusively: (1) initial renal Cd uptake is MT-independent and (2) retention of Cd in the kidney is MT-dependent. The later decrease in renal Cd in MT-null mice is evidently due to the inability of MT-null mice to synthesize “storage MT”. Yet the Nordbergs claimed the increased Cd-induced nephrotoxicity in MT-null mice is caused by deficient Cd-MT “transfer” from the liver to the kidneys (Nordberg and Nordberg 2025). This conclusion is illogical and far-fetching considering the enormous reduction of renal MT from 800 µg/g in wildtype to 0 µg/g in MT knockout mice (Liu et al. 1998) that cannot be accounted for by the uptake of extremely low concentrations of filtered “transport” (Cd-)MT (0.5–5 nM) derived from necrotic hepatocytes. Undoubtedly, renal Cd-MT in CLCE originates from Cd-induced “storage” MT and not from Cd-MT “transport” from the liver to the kidneys.

Evidently, there must be alternative nephrotoxic protein-bound Cd species. In a study by Fels et al*.* (Fels et al. 2019), toxicity of PT cells expressing megalin/cubilin was observed with 10 µM Cd-MT wheras Cd-MT at concentrations ≤ 1 µM were expectedly ineffective, which is compatible with the reported *K*_*d*_. In contrast, low Cd-β2M (100 nM), Cd-Alb (100 nM) and Cd-LCN2 (1 µM), but not Cd-alpha-1-microglobulin or Cd-Tf, induced cytotoxicity (Fels et al. 2019) (see also Suppl. Table 2). These concentrations are approximate to predicted concentrations of these proteins in the primary filtrate and *K*_*d*_ for megalin/cubilin: Alb ~ 0.6 µM, β2M ~ 0.4 µM, and LCN2 ~ 0.1 µM. Protein internalization was confirmed by uptake of fluorescently-labeled proteins. This crucial study reveals other Cd-protein complexes are more potent in eliciting PT damage than Cd-MT and further reiterates the unlikelihood of Cd-MT as the sole nephrotoxic Cd species.

### Where do the trace amounts of filtered Cd-MT go if they are not recovered by the PT?

Little MT is excreted in the urine therefore an alternative recovery mechanism must exist in the kidney. In mice perfused with ^109^CdCl_2_ or ^109^Cd-MT for 2–3 days, resulting in a steady-state plasma Cd concentration of 0.07 ng/ml (Johnson and Foulkes 1980), analogous to “normal” diets, preferential accumulation of Cd-MT in the kidney cortex was observed, but they did not clarify the localization of uptake, accumulation and toxicity of these low Cd-MT concentrations, as observed in CLCE. Indeed, a small but significant proportion of filtered proteins is reabsorbed by the distal segments of the nephron, both in Cd-exposed animals and workers (reviewed in (Thévenod and Wolff 2016)). Investigation of distal nephron injury has been neglected. When the PT is damaged (as in *Fanconi* syndrome) or uptake capacity is exceeded (e.g. in diabetes or hypertension), downstream nephron segments compensate for defective PT protein reabsorption. Indeed, human renal medulla accumulates up to 50% of Cd and (Cd-)MT levels in the cortex; Cd-inducible MT has been detected by immunohistochemistry in distal segments of the nephron (reviewed in (Thévenod and Wolff 2016)). In the distal nephron, the SLC22A17 receptor binds and endocytoses Cd-MT and other Cd-binding proteins (Langelueddecke et al. 2012) with much higher affinities (*K*_*d*_ ~ 100 nM) than those of megalin/cubilin, aligning with expected luminal protein concentrations (Thévenod et al. 2023) (see Suppl. Table 2B for details).

## Public health relevance of the current CLCE problem

CLCE causes chronic diseases and increases overall mortality because Cd has a very long biological half-life (> 20–30 years) and accumulates in organs, particularly in the kidneys, which may result in fibrosis, failure or, as a class 1 human carcinogen, cancer (Thévenod and Lee 2013). Hence, Cd exposure during childhood may affect health in old age. Co-morbidities and nephrotoxicity due to environmental pollution are thought to potentiate renal deterioration. In light of an ageing global population, Cd exposure may potentiate renal damage induced by other risk factors and co-morbidities, such as diabetes mellitus, cardiovascular or chronic inflammatory diseases, and vice versa, i.e. increased filtration of Cd-protein complexes in damaged kidneys (Madrigal et al. 2019). Cd accumulation and toxicity on top of these underlying diseases could worsen renal function in other segments and accelerate renal damage.

## How can a more accurate model of Cd nephrotoxicity be obtained?

This is an urgent need to drastically review and revise the outdated Nordberg model (compare Figs. 1A and B). The following issues need to be addressed for a more unbiased and fact-based model of chrCd-NTX:

1. What are the CLCE-relevant plasma Cd-complexes (protein, organic and inorganic molecules, others)? State-of-the-art analytical methodologies, such as X-ray diffraction, X-ray fluorescence, ICP-MS coupled with gel electrophoresis, which are commonly used in metalloproteomics research, are needed for reliable measurements.
2. Strikingly, no experimental evidence supports the claim for Cd-Alb uptake and leakage of generated Cd-MT from hepatocytes. The pertinent question remains as to why do plasma (Cd-)MT levels rise after Cd treatment? If damaged hepatocytes are not involved, release of Cd-MT by Kupffer cells and liver endothelial cells is plausible (enterocytes or pneumocytes could also produce and release (Cd-)MTs after Cd exposure). Thus, the role of hepatic cells in mediating uptake of plasma Cd/Cd-protein complexes must be clarified.
3. Kidney-specific knockout mice for PT megalin/cubilin and distal nephron SLC22A17 are available and should be used to investigate contribution of PT and distal nephron receptors in endocytosis and toxicity of filtered Cd-binding proteins in an adequate model of CLCE (Thijssen et al. 2007).

## Final thoughts

Since the 1950s, the world is changed; in its water, earth and air, which are determinant to how common individuals are exposed to Cd over decades, particularly in hotspots spread across Europe and Asia. Yet concepts in chrCd-NTX have stagnated and become anachronistic, choosing to ignore glaring omissions, blatant correlations and dismiss newer insightful studies. Scientific research on the mechanisms of Cd (nephro-)toxicity remains a timely and highly relevant issue of public health, both in newly industrialized countries and developed countries with aging populations.

The current model of Cd nephrotoxicity (Nordberg and Nordberg 2025) does not reflect demographic developments with co-morbidities that also affect renal function in aging populations on top of Cd exposure. Furthermore, extended life expectancies are strongly linked to changes in plasma composition thus potentially affecting Cd speciation and the mode of Cd transport in blood. Consequently, clarification of chrCd-NTX due to CLCE will serve to reduce end-stage renal disease and advocate awareness of global environmental health issues. By adopting a more open mindset and addressing the issues outlined above, the model of chrCd-NTX will undergo a renaissance and this will finally move the field forward.

## Supplementary Information

Below is the link to the electronic supplementary material.

Supplementary file1 (DOCX 24 KB)

Supplementary file2 (DOCX 25 KB)

## Data Availability

No original data were included in this COMMENTARY. The authors exclusively refer to published literature.
