# Supplementary material for: Challenging the current dogma of chronic Cd nephrotoxicity: myths and facts
Source: Arch Toxicol. 2026 Jan 14;100(4):1629–34. doi: 10.1007/s00204-025-04264-0 (PMC13043543; doi:10.1007/s00204-025-04264-0)
Supplement: Supplementary file 1 — Supplementary file1 (DOCX 24 KB) [file 204_2025_4264_MOESM1_ESM.docx]

**Suppl. Table 1:** Plasma concentrations of Cd-binding proteins and their estimated Cd binding affinities.

| **Ligand** | **Plasma concentration (µmol/l)** | **Refs** | ***K_D_* (mol/l))** | **Refs** |
| --- | --- | --- | --- | --- |
| MT | 0.0005-0.005 | (Milnerowicz and Bizon 2010) | ~10^-14^ | (Freisinger and Vasak 2013) |
| α2M | ~3.5 | (Yoshino et al. 2019) | ~10^-6^ | (Carson 1984) |
| β2M | 0.11 | (Norden et al. 2001) | n. a. | Ø |
| LCN2 | ~6.5 | (Magnusson et al. 2012) | n. a. | Ø |
| Alb | ~690 | (Norden et al. 2001) | ~10^-5^ | (Goumakos et al. 1991) |
| Tf | ~35.0 | (Norden et al. 2001) | ~10^-6^ | (Harris and Madsen 1988) |

**Abbreviations:** α2M = alpha-2 macroglobulin; Alb = albumin; β2M = beta-2 microglobulin; *K_D_* = dissociation constant; LCN2 = lipocalin-2; MT = metallothionein; n. a. = not available; Refs = references; Tf = transferrin.

Median Cd concentrations in blood of non-smoking populations amount to ~2-3 nmol/l (Elinder et al. 1983). Less than 10% of blood Cd is distributed in plasma (reviewed in (Thévenod and Lee 2013)).

**References**

Carson SD (1984) Cadmium binding to human alpha 2-macroglobulin. Biochim Biophys Acta 791(3):370-4 doi:10.1016/0167-4838(84)90349-2

Elinder CG, Friberg L, Lind B, Jawaid M (1983) Lead and cadmium levels in blood samples from the general population of Sweden. Environ Res 30(1):233-53 doi:10.1016/0013-9351(83)90183-4

Freisinger E, Vasak M (2013) Cadmium in metallothioneins. Met Ions Life Sci 11:339-371 doi:10.1007/978-94-007-5179-8_11

Goumakos W, Laussac JP, Sarkar B (1991) Binding of cadmium(II) and zinc(II) to human and dog serum albumins. An equilibrium dialysis and 113Cd-NMR study. Biochem Cell Biol 69:809-820

Harris WR, Madsen LJ (1988) Equilibrium studies on the binding of cadmium(II) to human serum transferrin. Biochemistry 27:284-288

Magnusson NE, Hornum M, Jorgensen KA, et al. (2012) Plasma neutrophil gelatinase associated lipocalin (NGAL) is associated with kidney function in uraemic patients before and after kidney transplantation. BMC nephrology 13:8 doi:10.1186/1471-2369-13-8

Milnerowicz H, Bizon A (2010) Determination of metallothionein in biological fluids using enzyme-linked immunoassay with commercial antibody. Acta biochimica Polonica 57:99-104

Norden AG, Lapsley M, Lee PJ, et al. (2001) Glomerular protein sieving and implications for renal failure in Fanconi syndrome. Kidney Int 60:1885-1892 doi:10.1046/j.1523-1755.2001.00016.x

Thévenod F, Lee WK (2013) Toxicology of cadmium and its damage to Mammalian organs. Met Ions Life Sci 11:415-490 doi:10.1007/978-94-007-5179-8_14

Yoshino S, Fujimoto K, Takada T, et al. (2019) Molecular form and concentration of serum alpha(2)-macroglobulin in diabetes. Scientific reports 9(1):12927 doi:10.1038/s41598-019-49144-7
