# Supplementary material for: Challenging the current dogma of chronic Cd nephrotoxicity: myths and facts
Source: Arch Toxicol. 2026 Jan 14;100(4):1629–34. doi: 10.1007/s00204-025-04264-0 (PMC13043543; doi:10.1007/s00204-025-04264-0)
Supplement: Supplementary file 2 — Supplementary file2 (DOCX 25 KB) [file 204_2025_4264_MOESM2_ESM.docx]

**Suppl. Table 2A:** Plasma concentrations, estimated concentrations in renal glomerular filtrate based on their calculated glomerular sieving coefficients (GSC), and binding affinities of LMWP and HMWP ligands of megalin (LRP2)/cubilin.

| **Ligand** | **Plasma conc. (nmol/l)** | **Molecular mass (kDa)** | **GSC^1^** | **Conc. in glomerular filtrate (nmol/l)** | ***K_D_ / EC_50_* (nmol/l))** |
| --- | --- | --- | --- | --- | --- |
| MT | 0.5-5 | 6.5 | ~0.9-1.0 | ~0.5-5 | 100,000 |
| β2M | 140 | 11.6 | ~0.9 | ~130 | 420 |
| LCN2 | 6,500 | 25 | ~0.2 | ~1,300 | 60 |
| Alb | 690,000 | 65.5 | <0.0001^4^ | 53 | 630 |
| Tf | 35,000 | 78 | <0.0001^4^ | 2 | 20 |

**Suppl. Table 2B:** Plasma concentrations, estimated concentrations in renal glomerular filtrate based on their calculated glomerular sieving coefficients (GSC), and binding affinities of LMWP and HMWP ligands of SLC22A17.

| **Ligand** | **Plasma conc. (nmol/l)** | **Molecular mass (kDa)** | **GSC^1^** | **Conc. in glomerular filtrate (nmol/l)^2^** | ***K_D_ / EC_50_* (nmol/l))** |
| --- | --- | --- | --- | --- | --- |
| MT | 0.5-5 | 6.5 | ~0.9-1.0 | ~0.5-5 | 123 ± 50 |
| β2M | 140 | 11.6 | ~0.9 | ~130 | n.d. |
| LCN2 | 6,500 | 25 | ~0.2 | ~1,300 | 0.092 ± 0.006 |
| Alb | 690,000 | 65.5 | <0.0001^4^ | 53 | ***10-2,800^3^*** |
| Tf | 35,000 | 78 | <0.0001^4^ | 2 | ***350-2,800^3^*** |

Abbreviations: Alb = albumin; β2M = beta-2 microglobulin; *EC_50_* = Half maximal effective concentration; HMWP = high-molecular weight proteins; *K_D_* = dissociation constant; LCN2 = lipocalin-2; LMWP = low-molecular weight proteins; LRP2 = Low-Density Lipoprotein Receptor-Related Protein 2; MT = metallothionein; Tf = transferrin.

Data in tables are summarized from information reviewed in references (Fels et al. 2019; Thévenod et al. 2023; Thévenod and Wolff 2016).

^1^: GSC was calculated according to: GSC = C_Filtrate_ / C_Plasma_ (Maack et al. 1992).

^2^: In the distal nephron where SLC22A17 is expressed, luminal solute concentrations
increase 15-100-fold consequent to fluid reabsorption (Ullrich 1975).

^3^: Concentration range tested to saturation (Langelueddecke et al. 2012).

**References**

Fels J, Scharner B, Zarbock R, et al. (2019) Cadmium Complexed with beta2-Microglubulin, Albumin and Lipocalin-2 rather than Metallothionein Cause Megalin:Cubilin Dependent Toxicity of the Renal Proximal Tubule. International journal of molecular sciences 20(10) doi:10.3390/ijms20102379

Langelueddecke C, Roussa E, Fenton RA, Wolff NA, Lee WK, Thévenod F (2012) Lipocalin-2 (24p3/neutrophil gelatinase-associated lipocalin (NGAL)) receptor is expressed in distal nephron and mediates protein endocytosis. J Biol Chem 287:159-169

Maack T, Park CH, Camargo MJF (1992) Renal filtration, transport and metabolism of proteins. In: Seldin DW, Giebisch G (eds) The Kidney: Physiology and Pathophysiology. Raven, New York, p 3005-3038

Thévenod F, Herbrechter R, Schlabs C, et al. (2023) Role of the SLC22A17/lipocalin-2 receptor in renal endocytosis of proteins/metalloproteins: a focus on iron- and cadmium-binding proteins. Am J Physiol Renal Physiol 325(5):F564-F577 doi:10.1152/ajprenal.00020.2023

Thévenod F, Wolff NA (2016) Iron transport in the kidney: implications for physiology and cadmium nephrotoxicity. Metallomics : integrated biometal science 8:17-42

Ullrich KJ (1975) Niere. In: Keidel WD (ed) Kurzgefaßtes Lehrbuch der Physiologie. Georg Thieme Verlag, Stuttgart
